# MetChem: a new pipeline to explore structural similarity across metabolite modules

**DOI:** 10.1093/bioadv/vbad053

**Published:** 2023-04-21

**Authors:** Ebtesam A Abdel-Shafy, Tadele Melak, David A MacIntyre, Giorgia Zadra, Luiz F Zerbini, Silvano Piazza, Stefano Cacciatore

**Affiliations:** Bioinformatics Unit, International Centre for Genetic Engineering and Biotechnology, Cape Town 7925, South Africa; National Research Centre, Cairo 12622, Egypt; Computation Biology, International Centre for Genetic Engineering and Biotechnology, Trieste 34149, Italy; Department of Clinical Chemistry, University of Gondar, Gondar 196, Ethiopia; March of Dimes Prematurity Research Centre, Imperial College London, London SW7 2AZ, UK; Imperial College Parturition Research Group, Institute of Reproductive and Developmental Biology, Department of Metabolism, Digestion and Reproduction, Imperial College London, London SW7 2AZ, UK; Institute of Molecular Genetics, National Research Council, Pavia 27100, Italy; Cancer Genomics, International Centre for Genetic Engineering and Biotechnology, Cape Town 7925, South Africa; Computation Biology, International Centre for Genetic Engineering and Biotechnology, Trieste 34149, Italy; Bioinformatics Unit, International Centre for Genetic Engineering and Biotechnology, Cape Town 7925, South Africa; Imperial College Parturition Research Group, Institute of Reproductive and Developmental Biology, Department of Metabolism, Digestion and Reproduction, Imperial College London, London SW7 2AZ, UK

## Abstract

**Summary:**

Computational analysis and interpretation of metabolomic profiling data remains a major challenge in translational research. Exploring metabolic biomarkers and dysregulated metabolic pathways associated with a patient phenotype could offer new opportunities for targeted therapeutic intervention. Metabolite clustering based on structural similarity has the potential to uncover common underpinnings of biological processes. To address this need, we have developed the MetChem package. MetChem is a quick and simple tool that allows to classify metabolites in structurally related modules, thus revealing their functional information.

**Availabilityand implementation:**

MetChem is freely available from the R archive CRAN (http://cran.r-project.org). The software is distributed under the GNU General Public License (version 3 or later).

## 1 Introduction

Metabolomics is a post-genomic research field that consists in the unbiased analysis of small molecule metabolites in biological samples such as tissue specimens (Priolo *et al.*, 2014), cell lines (Zadra *et al.*, 2019) and biofluids (Aimetti *et al.*, 2012; Bertini *et al.*, 2012). Each cell type or biofluid is characterized by unique metabolic signature, which can be altered in pathophysiological conditions (Cacciatore and Loda, 2015; Cacciatore *et al.*, 2021). Metabolic concentrations are regulated by complex biological pathways, of which enzymes are key modulators. Despite their known specificity, many enzymes are reported to have promiscuous activity and catalyze reactions of molecules that are chemically like their specific substrates (Piedrafita *et al.*, 2015). Pathological events can also change the concentration of specific molecule classes in non-enzymatic manner, such as through mechanical occlusion (Elebo *et al.*, 2021). During both biological activity and preanalytical procedures, non-enzymatic reactions can also muddle the structure and concentration of metabolites that either belong to the same chemical class or harbor specific functional groups (Cacciatore *et al.*, 2013, 2017a). Metabolites with a similar chemical structure tend to share similar properties and biological activity (Ekaney *et al.*, 2021; Menikarachchi *et al.*, 2013).

Most conventional computational tools for metabolite mapping and enrichment analysis rely primarily on a wide variety of curated pathway databases (Banimfreg *et al.*, 2022; Pang *et al.*, 2021). Unfortunately, the coverage of currently annotated pathways in the human metabolic network remains poor. Recently, chemical similarity has been used to extend known metabolic pathways to include unreported metabolites (Grapov *et al.,* 2015; Pertusi *et al.* 2015).

The first study that applied structural similarity to classifying *Escherichia coli* metabolome into chemically and functionally related groups was reported by Nobeli *et al.* (2003). After this, there have been many attempts to develop computer aided tools that integrate chemical structure into metabolite analysis and visualization such as MetaMapR (Grapov *et al.*, 2015) and ChemRICH (Barupal and Fiehn, 2017). However, conventional algorithms fail to identify local clusters of metabolites with similar structures (Andronov *et al.*, 2021). Thus, there is still an unmet need for tools that can identify chemical similarity cluster with high accuracy.

Here, we present *MetChem*, a new R package built on the KODAMA, an unsupervised machine learning algorithm for dimensionality reduction that has been successfully applied for clustering identification (Cacciatore *et al.*, 2014, Zinga *et al.*, 2023). *MetChem* is designed to provide metabolomics researchers with a user-friendly pipeline able to investigate features in metabolic modules defined by their structural similarity. *MetChem* is also equipped with different functions to provide identified clusters with information from the Human Metabolite Database (HMDB) (Wishart *et al.*, 2022).

## 2 Features and methods

### 2.1 Data input

The MetChem pipeline requires as input the metabolites’ concentrations of each sample, the simplified molecular-input line-entry system (SMILES) and the HMDB identifiers associated to each chemical name. These can be retrieved from PubChem Identifier Exchange Service (https://pubchem.ncbi.nlm.nih.gov/idexchange/idexchange.cgi).

### 2.2 Metabolite structural clustering

**Fig. 1.**
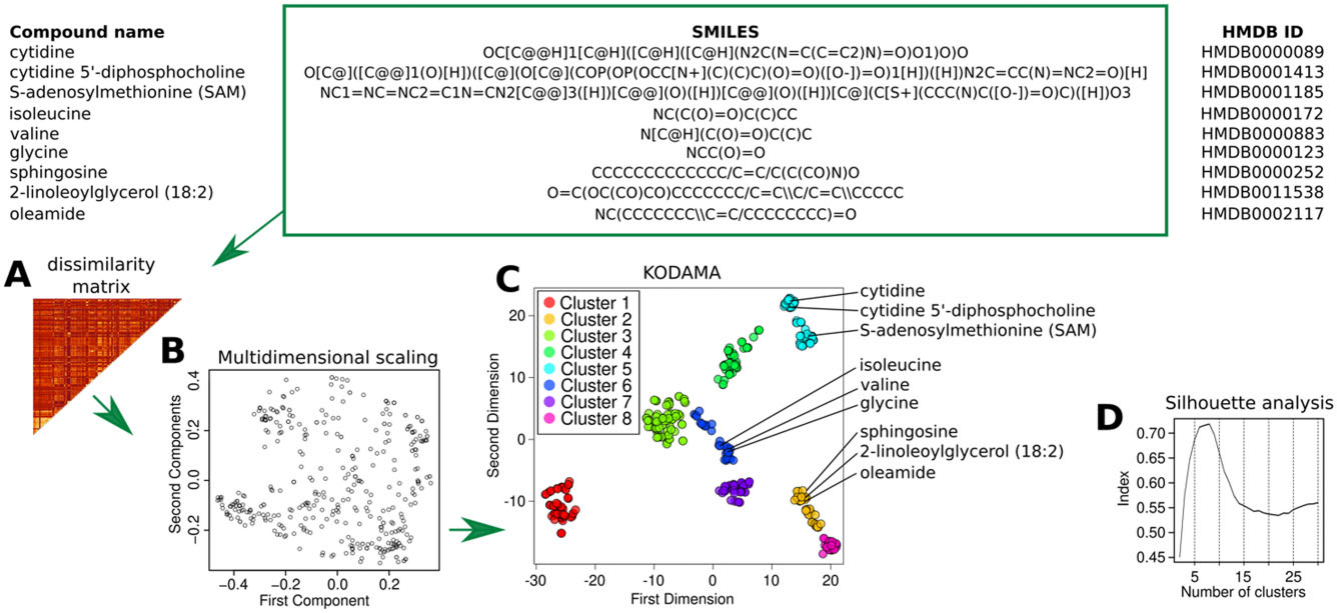
Metabolite clustering based on chemical similarity. In the first step, (**A**) SMILES are converted in a molecular fingerprint. Tanimoto distance is used to produce a dissimilarity matrix between metabolites. (**B**) Multidimensional scaling is applied to convert the dissimilarity matrix in a multidimensional space, with 50 dimensions as default. (**C**) KODAMA analysis is performed on the output of the multidimensional scaling to highlight the chemical similarity between metabolites. (**D**) The optimal number of clusters representing different chemical classes is defined by Rousseeuw’s Silhouette quality index

As the first step in the pipeline, the *clusters.detection* function uses the molecular structure of metabolites represented by the SMILES is used to visualize in a two-dimensional space the chemical similarity across metabolites and, subsequently, to identify the local clusters (Fig. 1). This function converts SMILES into molecular fingerprints, encoding their structural characteristics as a vector. The distance between two metabolites is calculated using a distance method such as Tanimoto to produce a dissimilarity matrix (Fig. 1A). This dissimilarity matrix is then converted into a multidimensional space (Fig. 1B) prior to being processed by KODAMA, using the homonym package (Cacciatore *et al.*, 2017b). The output generated by KODAMA is a two-dimensional plot representing the ‘chemical distances’ between all metabolites (Fig. 1C). Metabolite hierarchical clustering is then built on the KODAMA output. Rousseeuw’s Silhouette quality index (Rousseeuw, 1987) is used to calculate the optimal number of clusters.

To ensure the reproducibility of the results, the entire procedure is repeated (10 times as default) and average Rousseeuw’s Silhouette quality index values are calculated (Fig. 1D).

### 2.3 Weighted metabolite chemical similarity analysis

**Fig. 2.**
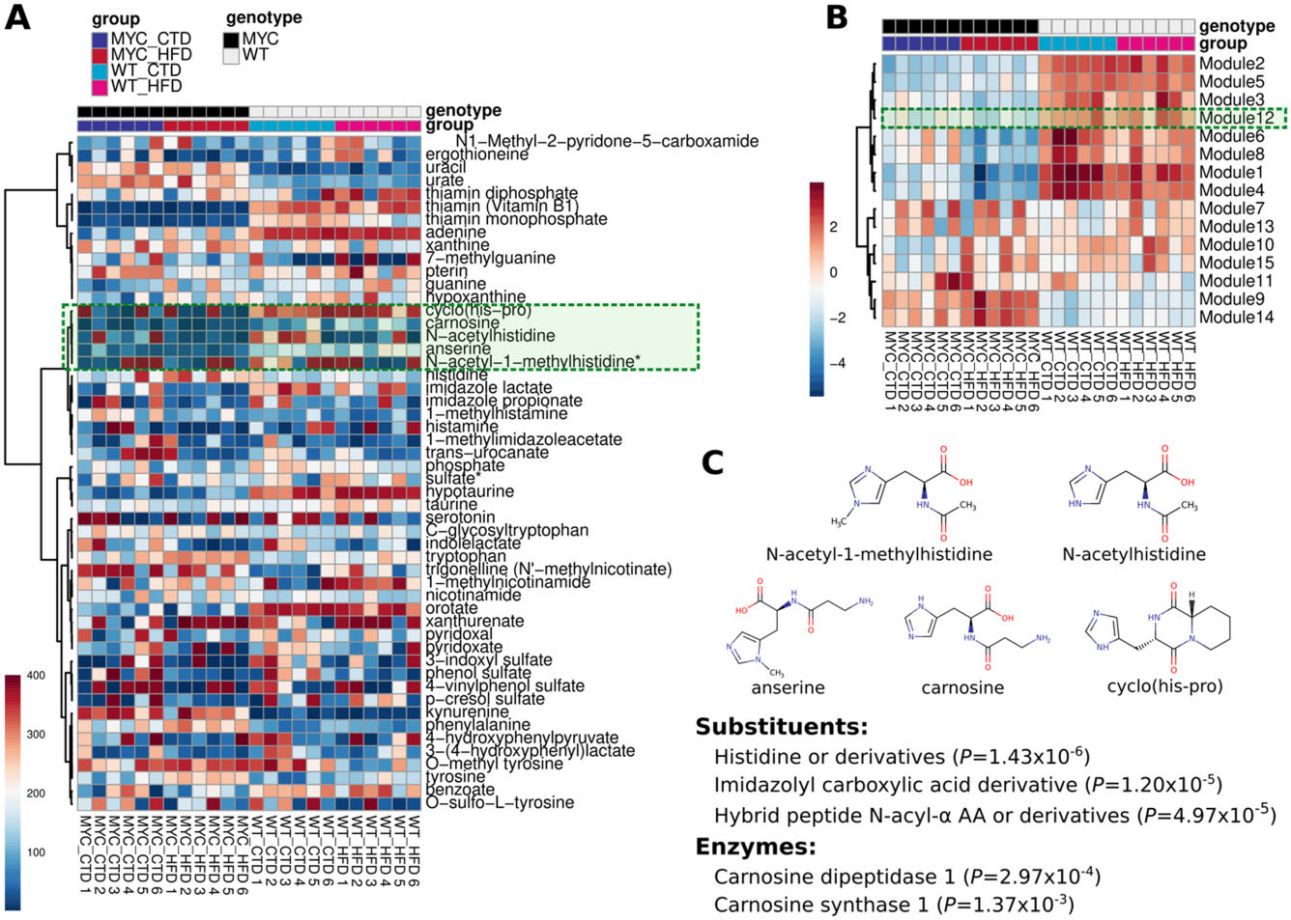
Functional enrichment analysis. (**A**) Heatmap of metabolites belonging to a cluster identified by the *clusters.detection* function. The metabolites are clustered based on their chemical structures using the KODAMA outputs. (**B**) All different metabolite subclasses (a.k.a. modules) are identified by cutting the tree using the *allbranches* function. Weighted Metabolite Chemical Similarity Analysis (WMCSA) is performed on all identified modules. (**C**) Chemical and biological information of each module can be retrieved from HMDB using *readMet* function

The weighted gene co-expression network analysis (WGCNA) is a method that was originally developed not only to quantify the correlation between a pair of genes, but also to determine the extent of shared neighbors and define topological overlap in microarray dataset (Langfelder and Horvath, 2008). Recently, WGCNA has been also applied to metabolomic research (Mock *et al.*, 2018). Akin to WGCNA, we have developed the Weighted Metabolite Chemical Similarity Analysis (WMCSA). In this pipeline, WMCSA is applied to a selected cluster of metabolites identified by the *clusters.detection* function (Fig. 2A). WMCSA is implemented in the homonym function *WMCSA*. This function summarizes metabolite concentrations in modules that are defined by metabolite chemical similarity. Modules are generated by cutting all possible different branches of the hierarchical clustering performed on the selected class of metabolites. Each module is represented by one hypothetical vector called ‘module eigen metabolite’, which is equivalent to the first component of the principal component analysis (Fig. 2B).

### 2.4 HMDB data retrieval and feature analysis

In HMDB, the metabolite structural and functional information is stored as a so called ‘metabocard’ where each metabocard contains more than hundred descriptors of biological, clinical and physical-chemical data hyperlinked to other databases (e.g. KEGG, PubChem, MetaCyc, ChEBI).

The *MetChem* package includes functions to download, extract and process information from HMDB. The function *readMet* gives full access to metabolite descriptors that are stored as individual metabocards in the HMDB and convert them into an accessible data format for R users. The functions *nameMet*, *propertiesMet*, *substituentsMet* and *taxonomyMet*, *EnzymesMet*, *diseasesMet* and *pathwaysMet* extract names, chemical properties, substituents, taxonomy, enzymes, disease and pathways for all metabolites in each module.

After retrieval of the relevant information from HMDB, each module is associated with chemical and biological attributes. The function *features* compare the different attributes of each metabolite according to their belonging to each module (Fig. 2C). The final output of the *MetChem* pipeline is a summary of metabolite data in different modules with defined chemical and biological properties.

## 3 Case study

The *MetChem* pipeline was applied to a metabolomic dataset from the study by Labbé *et al.* (2019). The dataset contains the metabolic profiles from ventral prostate tissues of mice that overexpress the human c-MYC transgene (MYC) in the prostate epithelium and wild-type littermates (WT). Mice were fed either a high fat diet (HFD; 60% kcal from fat; lard-rich in saturated fat) or a control diet (CTD; 10% kcal from fat) for 9 weeks. The dataset includes six replicates for each group (i.e. WT_CTD, MYC_CTD, WT_HFD and MYC_HFD). A total of 398 metabolites were associated with their own SMILES representation. In this dataset, eight different chemical classes were identified (Fig. 1C and D). A WMCSA was performed in one selected chemical class as representative example to generate module representation (Fig. 2B). Statistically significant different modules between MYC and WT were identified. The module 12 was chosen for further inspection. The module consists of the metabolites anserine, carnosine, cyclo(his-pro), N-acetylhistidine and N-acetyl-1-methylhistidine, which are characterized by the presence of common significant substituents, such as imidazolyl carboxylic acid. Related enzymes were also identified (Fig. 2C). Although, the conversion of methylhistidine from anserine and carnosine has been described as indication of muscle breakdown (Hsieh *et al.*, 2022), the relation with cyclo(his-pro), N-acetylhistidine and N-acetyl-1-methylhistidine has not been characterized yet, which opens opportunities for further investigation. We observed that reduced concentration of these metabolites was associated with the overexpression of MYC, a well-known oncogene involved in the regulation of the glycolysis (Dang, 2012). Since carnosine exerts metabolism-dependent effects, including potential inhibition of glycolysis (Gaunitz and Hipkiss, 2012), we speculate that the metabolic effects of carnosine reduction in the MYC-transformed prostate may also involve other imidazolyl carboxylic acids of the module, which is warrant further investigation.

## 4 Summary and outlook

*MetChem* allows integrating chemical and concentration information in the analysis of metabolomic data. Thus, *MetChem* may provide important biological insights in pathological and physiological conditions.

## References

[vbad053-B1] Andronov M. et al (2021) Exploring chemical reaction space with reaction difference fingerprints and parametric t-SNE. ACS Omega, 6, 30743–30751. 34805702 10.1021/acsomega.1c04778 PMC8600617

[vbad053-B2] Aimetti S. et al (2012) Metabonomic analysis of saliva reveals generalized chronic periodontitis signature. Metabolomics, 8, 465–474.

[vbad053-B3] Banimfreg B.H. et al (2022) Survey for computer-aided tools and databases in metabolomics. Metabolites, 12, 1002. 36295904 10.3390/metabo12101002 PMC9610953

[vbad053-B4] Barupal D.K. , Fiehn O. (2017) Chemical similarity enrichment analysis (ChemRICH) as alternative to biochemical pathway mapping for metabolomic datasets. Sci. Rep., 7, 1–11. 29109515 10.1038/s41598-017-15231-w PMC5673929

[vbad053-B5] Bertini I. et al (2012) Metabolomic NMR fingerprinting to identify and predict survival of patients with metastatic colorectal cancer. Cancer Res., 72, 356–364. 22080567 10.1158/0008-5472.CAN-11-1543

[vbad053-B6] Cacciatore S. , Loda M. (2015) Innovation in metabolomics to improve personalized healthcare. Ann. N. Y. Acad. Sci., 1346, 57–62. 26014591 10.1111/nyas.12775 PMC4839187

[vbad053-B7] Cacciatore S. et al (2013) Effects of intra- and post-operative ischemia on the metabolic profile of clinical liver tissue specimens monitored by NMR. J. Proteome Res., 12, 5723–5729. 24124761 10.1021/pr400702d

[vbad053-B8] Cacciatore S. et al (2014) Knowledge discovery by accuracy maximization. Proc. Natl. Acad. Sci. USA, 111, 5117–5122. 24706821 10.1073/pnas.1220873111 PMC3986136

[vbad053-B9] Cacciatore S. et al (2017a) Profiling in formalin-fixed and paraffin-embedded prostate cancer tissues. Mol. Cancer Res., 15, 439–447. 28074002 10.1158/1541-7786.MCR-16-0262 PMC5552883

[vbad053-B10] Cacciatore S. et al (2017b) KODAMA: an R package for knowledge discovery and data mining. Bioinformatics, 33, 621–623. 27993774 10.1093/bioinformatics/btw705 PMC5408808

[vbad053-B11] Cacciatore S. et al (2021) Inflammatory metabolic profile of South African patients with prostate cancer. Cancer Metab., 9, 1–14. 34344464 10.1186/s40170-021-00265-6 PMC8336341

[vbad053-B12] Dang C.V. (2012) MYC on the path to cancer. Cell, 149, 22–35. 22464321 10.1016/j.cell.2012.03.003 PMC3345192

[vbad053-B13] Ekaney L.Y. et al (2021) Chemical similarity methods for analyzing secondary metabolite structures. Phys. Sci. Rev., 6, 247–264.

[vbad053-B14] Elebo N. et al (2021) Metabolomic and lipoprotein profiling of pancreatic ductal adenocarcinoma patients of African ancestry. Metabolites, 11, 663. 34677378 10.3390/metabo11100663 PMC8540259

[vbad053-B16] Gaunitz F. , Hipkiss A.R. (2012) Carnosine and cancer: a perspective. Amino Acids, 43, 135–142. 22454085 10.1007/s00726-012-1271-5

[vbad053-B700] Grapov D. et al (2015) MetaMapR: pathway independent metabolomic network analysis incorporating unknowns. Bioinformatics, 31, 2757–2760. 25847005 10.1093/bioinformatics/btv194 PMC4528626

[vbad053-B17] Hsieh S.L. et al (2022) Carnosine suppresses human colorectal cancer cell proliferation by inducing necroptosis and autophagy and reducing angiogenesis. Oncol. Lett., 23, 44. 34976156 10.3892/ol.2021.13162 PMC8674876

[vbad053-B18] Labbé D.P. et al (2019) High-fat diet fuels prostate cancer progression by rewiring the metabolome and amplifying the MYC program. Nat. Commun., 10, 1–14. 31554818 10.1038/s41467-019-12298-z PMC6761092

[vbad053-B19] Langfelder P. , Horvath S. (2008) WGCNA: an R package for weighted correlation network analysis. BMC Bioinformatics, 9, 559. 19114008 10.1186/1471-2105-9-559 PMC2631488

[vbad053-B20] Menikarachchi L.C. et al (2013) Chemical structure identification in metabolomics: computational modeling of experimental features. Comput. Struct. Biotechnol. J., 5, e201302005. 24688698 10.5936/csbj.201302005 PMC3962140

[vbad053-B21] Mock A. et al (2018) MetaboDiff: an R package for differential metabolomic analysis. Bioinformatics, 34, 3417–3418. 29718102 10.1093/bioinformatics/bty344 PMC6157071

[vbad053-B249] Nobeli,I. et al (2003) A structure-based anatomy of the E.coli metabolome. J. Mol. Biol., 334, 697–719. 14636597 10.1016/j.jmb.2003.10.008

[vbad053-B22] Pang Z. et al (2021) MetaboAnalyst 5.0: narrowing the gap between raw spectra and functional insights. Nucleic Acids Res., 49, W388–W396. 34019663 10.1093/nar/gkab382 PMC8265181

[vbad053-B23] Pertusi D.A. et al (2015) Efficient searching and annotation of metabolic networks using chemical similarity. Bioinformatics, 31, 1016–1024. 25417203 10.1093/bioinformatics/btu760 PMC4382900

[vbad053-B24] Piedrafita G. et al (2015) The impact of non-enzymatic reactions and enzyme promiscuity on cellular metabolism during (oxidative) stress conditions. Biomolecules, 5, 2101–2122. 26378592 10.3390/biom5032101 PMC4598790

[vbad053-B25] Priolo C. et al (2014) AKT1 and MYC induce distinctive metabolic fingerprints in human prostate cancer. Cancer Res., 74, 7198–7204. 25322691 10.1158/0008-5472.CAN-14-1490 PMC4267915

[vbad053-B26] Rousseeuw P.J. (1987) Silhouettes: a graphical aid to the interpretation and validation of cluster analysis. J. Comput. Appl. Math., 20, 53–65.

[vbad053-B27] Wishart D.S. et al (2022) HMDB 5.0: the human metabolome database for 2022. Nucleic Acids Res., 50, D622–D631. 34986597 10.1093/nar/gkab1062 PMC8728138

[vbad053-B28] Zadra G. et al (2019) Inhibition of de novo lipogenesis targets androgen receptor signaling in castration-resistant prostate cancer. Proc. Natl. Acad. Sci. USA, 116, 631–640. 30578319 10.1073/pnas.1808834116 PMC6329966

[vbad053-B29] Zinga M.M. et al (2023) KODAMA exploratory analysis in metabolic phenotyping. Front. Mol. Biosci., 9, 1436. 10.3389/fmolb.2023.1165720 PMC10036897 36968275

